# Differences in the transactivation domains of p53 family members: a computational study

**DOI:** 10.1186/1471-2164-11-S1-S5

**Published:** 2010-02-10

**Authors:** Jagadeesh N Mavinahalli, Arumugam Madhumalar, Roger W Beuerman, David P Lane, Chandra Verma

**Affiliations:** 1 Bioinformatics Institute (A-STAR), 30 Biopolis Street, Matrix, Singapore 138671; 2 Singapore Eye Research Institute, 11 Third Hospital Avenue, Singapore 168751; 3 Institute of Molecular and Cell Biology (A-STAR), 61 Biopolis Drive, Proteos, Singapore 138673

## Abstract

The N terminal transactivation domain of p53 is regulated by ligases and coactivator proteins. The functional conformation of this region appears to be an alpha helix which is necessary for its appropriate interactions with several proteins including MDM2 and p300. Folding simulation studies have been carried out to examine the propensity and stability of this region and are used to understand the differences between the family members with the ease of helix formation following the order p53 > p73 > p63. It is clear that hydrophobic clusters control the kinetics of helix formation, while electrostatic interactions control the thermodynamic stability of the helix. Differences in these interactions between the family members may partially account for the differential binding to, and regulation by, MDM2 (and MDMX). Phosphorylations of the peptides further modulate the stability of the helix and control associations with partner proteins.

## Background

The tumour suppressor protein p53 is a transcription factor, important for the stress management of eukaryotic cells. Under cellular stress p53 activates pathways responsible for cell cycle arrest, DNA repair, senescence and apoptosis [1,2]. p53 partly belongs to the class of intrinsically unstructured proteins (IUP) [3], where certain domains get structured upon interaction with other partners. The N-terminal transactivation domain (TA) of p53 is intrinsically largely unstructured, and is the binding site for components of the transcription machinery like p300/CBP, TAFII40/60 and of negative regulators MDM2, MDMX [4-9]. Overexpression of MDM2/MDMX as is seen in some tumours [10] can inactivate p53 by degradation and hence lead to tumour survival. This makes this interaction a potential therapeutic target for interruption in an effort to stabilize and activate p53 [11]. p53 TA adopts an α-helical conformation upon binding to MDM2, MDMX [12] or to p300 [13]. NMR studies have shown that this domain lacks persistent structural order in solution in the absence of MDM2 or p300, except for a small region that remains helical [14]. Thus there is a strong coupling between binding and folding in the functional interactions of p53 and MDM2/MDMX/p300 [15]. The consensus view is that the ability of the TA region (residues 17-29) to adopt a helical conformation is linked to its interactions with partners. It is important to develop a detailed understanding of how this region folds and several studies have contributed towards this [16-18]. Peptides that have been engineered to induce higher helicity in this region such as the stapled peptides [19] or those with altered sequence [20,21] have been demonstrated to bind with more avidity than the native peptide [22].

The p53 family also consists of two homologues, p63 and p73. Their main functions have been shown to be critical for development and differentiation. p63 has been shown to be essential for limb, skin and craniofacial morphogenesis [23-27]; and p73 has been shown to be involved in regulation of both the stress response and development [28]. Phylogenetic analysis of these members suggests that p53 might have evolved from an ancestral p63/p73 like gene [29,30]. All the family members have similar domain architecture: an N-terminal transactivation domain (TAD), a central DNA binding domain (DBD) and a C-terminal oligomerisation domain (OD) [31,32]. The N-terminus is the least conserved domain among the family members (~25%-29% sequence identity). Interestingly, both p63 and p73 can activate sets of p53 target genes including MDM2 [30,33,34]. In addition, the three important residues (F19, W23 and L26, see Table 1), which are key players in the MDM2-p53 interaction, are conserved in p63 and p73; there is no structural data available on this region of p63 and p73. It is well known that p53 and p73 are regulated by MDM2 in a similar manner by interactions between their N-terminal domains [35]. However the interaction between the N-terminal domain of p63 and that of MDM2 has been controversial [36,37]. This is intriguing since all three sequences contain the sequence FXXXWXXL which is the motif recognized by the N-terminal domain of MDM2.

**Table 1:** Sequences of peptides simulated

	Peptides studied	17	18	***19***	20	21	22	***23***	24	25	***26***	27	28	29
1	P53	E	T	*F*	S	D	L	*W*	K	L	*L*	P	E	N
2	P63	E	V	*F*	Q	H	I	*W*	D	F	*L*	E	Q	P
3	P73	T	T	*F*	E	H	L	*W*	S	S	*L*	E	P	D
4	P53L22I	E	T	*F*	S	D	**I**	*W*	K	L	*L*	P	E	N
5	P63I22L	E	V	*F*	Q	H	**L**	*W*	D	F	*L*	E	Q	P
6	P73L22I	T	T	*F*	E	H	**I**	*W*	S	S	*L*	E	P	D
7	P63H21D_D24K	E	V	*F*	Q	**D**	I	*W*	**K**	F	*L*	E	Q	P
8	P73H21D_S24K	T	T	*F*	E	**D**	L	*W*	**K**	S	*L*	E	P	D
9	P53L25F	E	T	*F*	S	D	L	*W*	K	**F**	*L*	P	E	N
10	P63F25L	E	V	*F*	Q	H	I	*W*	D	**L**	*L*	E	Q	P
11	P73S25F	T	T	*F*	E	H	L	*W*	S	**F**	*L*	E	P	D
12	P63DLWK	E	V	*F*	Q	**D**	**L**	*W*	**K**	F	*L*	E	Q	P

Conserved residues are shown in *italics*. Difference to wild type residue is shown in **bold**.

In this study, we set out to explore the relationship between the nature of amino acids that constitute the 17-29 region (p53 numbering) of the TA domains in this family and their ability to fold into conformations that may modulate their interactions with MDM2; in related work the details of these interactions have been modelled [38]. We examine why MDM2 interacts preferentially with p53 and p73. In addition we attempt to distil features that may aid in the design of high affinity peptides that could be used to disrupt the MDM2-p53/p73 interactions as potential therapeutics [39].

## Methods

The initial linear peptide conformers were generated using the XLEAP module of AMBER9 [40]. MD simulations were carried out using an implicit solvent method (GB) that has been shown to be successful in simulating peptide folding [41]. The major advantage of this method over using explicit solvent is faster sampling which enables us to study several peptides. The force field ff96 was used along with the Onufriev, Bashford and Case model (incorporated in AMBER under the option igb=5) [42] for optimal Born radii for macromolecules. A salt concentration of 0.2Mm was used. Hydrogen containing bonds were constrained using SHAKE [43]. Simulations were carried out at 325K to explore larger regions of conformational space. After initial minimizations, the system was gradually heated to 325K, equilibrated for 100 ps and production runs were carried out for 100ns on the wild type sequences of p53/p63/p73 and several mutants; in addition, the effects of phosphorylation were also examined in p53/p73 (this stretch of p63 does not contain any S/T residues). A total of 20 peptides (listed in Table 1) was each simulated for up to 100ns – resulting in a total simulation time of 2μs. In addition, 80ns of explicit water simulations were carried out on structures that were taken from the implicit solvent simulations for p53, p63 and p73, using the TIP3P model [44]. Analysis was carried out using Visual Molecular Dynamics (VMD) Version 1.8.6 [45]. Cluster analysis was performed using kclust module of the MMTBSA tool set [46]. Grace was used to plot all the graphs. Hydrogen bond analysis was carried out using the ptraj module of Amber9 with a distance cut off of 3Å and an angle cut off of 120.0 degrees.

## Results and discussion

### Folding patterns in p53, p63 and p73

**Figure 1:**
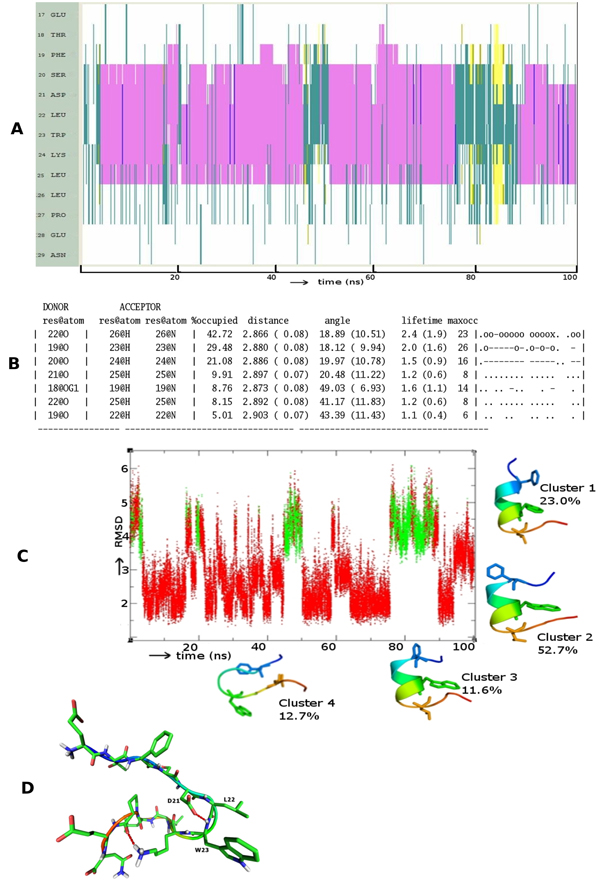
**Folding pattern of p53** (A) Evolution of secondary structures of the p53 peptides as a function of simulation time Colour code: purple, α-helix; red, π-helix; yellow, β-sheet; green, isolated bridge; cyan, turn; white, random coil. (B) Hydrogen bond statistics of the secondary structures averaged over 100 ns of simulations; the lifetime of hydrogen bonds in 5 ns windows is shown as: Space ( ) for 0-5%, dot (.) for 5-20%, dash (-) for 20-40%, o for 40-60%, x for 60-80%, star (*) for 80-95% and at (@) for 95 – 100%. (C) Cluster analysis of secondary structures in terms of RMSD as a function of simulation time; a representative structure (N-terminus in blue, C-terminus in red) from each cluster is shown with % of population;  colour code of the plot: red is helix, yellow is β-Sheet and green is random structure. Conserved residues F19, W23 and L26 are shown as sticks. (D) Snapshot of the putative nucleation conformation of p53during the folding simulation; nucleation residue L22 and hydrogen bond between D21 side chain and backbone of W23 are shown. Hydrogen Bonds are shown as red dotted lines.

Figure 1A shows that the region of p53 that embeds into MDM2 readily folds into a helix and is stable throughout the simulation. Although there are structural fluctuations, these are localized largely to the terminal residues, especially the C-terminus beyond P27. These fluctuations unwind the helix for short durations. Hydrogen bond (HB) analysis (Figure 1B) shows that a strong and long lived (42% occupancy) back bone hydrogen bond between L22 and E17 together with two other backbone HBs with 29% and 21% occupancy respectively between residues F19-W23 and S20-K24 stabilize the helix. Analysis of conformations sampled (Figure 1C) show four major clusters – three represent helices covering largely the region of p53 that embeds into MDM2 (and covers 86% of the conformational space) and a smaller cluster represents unstructured p53.

**Figure 2:**
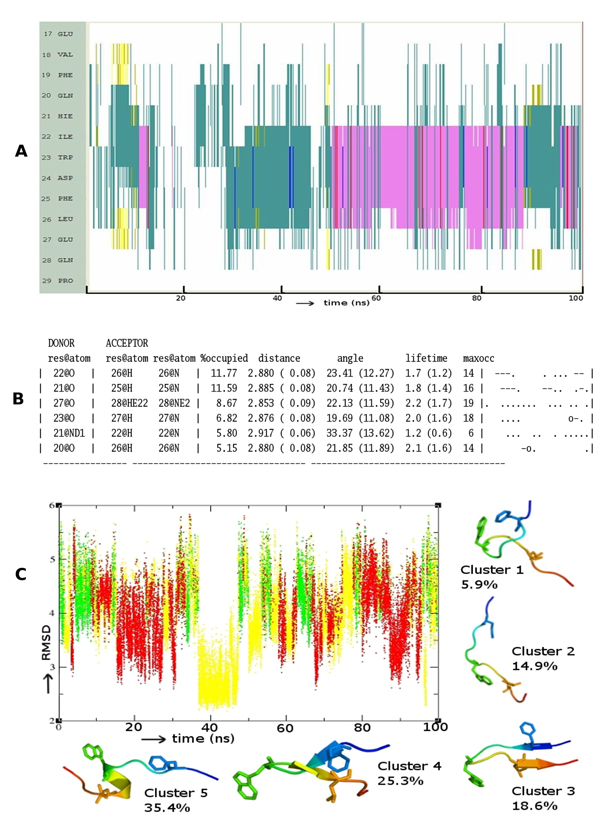
**Folding pattern of p63** (A) Evolution of secondary structures of the p63 peptides as a function of simulation time Colour code: purple, α-helix; red, π-helix; yellow, β-sheet; green, isolated bridge; cyan, turn; white, random coil. (B) Hydrogen bond statistics of the secondary structures averaged over 100 ns of simulations; the lifetime of hydrogen bonds in 5 ns windows is shown as: Space ( ) for 0-5%, dot (.) for 5-20%, dash (-) for 20-40%, o for 40-60%, x for 60-80%, star (*) for 80-95% and at (@) for 95 – 100%. (C) Cluster analysis of secondary structures in terms of RMSD as a function of simulation time; a representative structure (N-terminus in blue, C-terminus in red) from each cluster is shown with % of population;  colour code of the plot: red is helix, yellow is β-Sheet and green is random structure. Conserved residues F19, W23 and L26 are shown as sticks.

In contrast to p53, it is clear from Figure 2A that p63 takes a relatively long time to fold into an α-helix and this in turn does not seem to be very stable, unfolding frequently to a disordered state. There is no evidence of helicity at the ends of the peptide and when the helix does form, it begins at H21 (the region of the TA that needs to be helical for appropriate interactions with MDM2 begins at around residue 18). Figure 2B clearly shows that most HBs are transient with the exception of two backbone HBs (between residues L22-L26 and D21-L25) with occupancy of around 11%. Figure 2C shows two clusters of unstructured motifs: two in β-sheet and a single cluster representing a helical motif. Although 34% of the structures are helical, we find that they are short both in space and in time and transit rapidly between helical and non-helical states.

**Figure 3:**
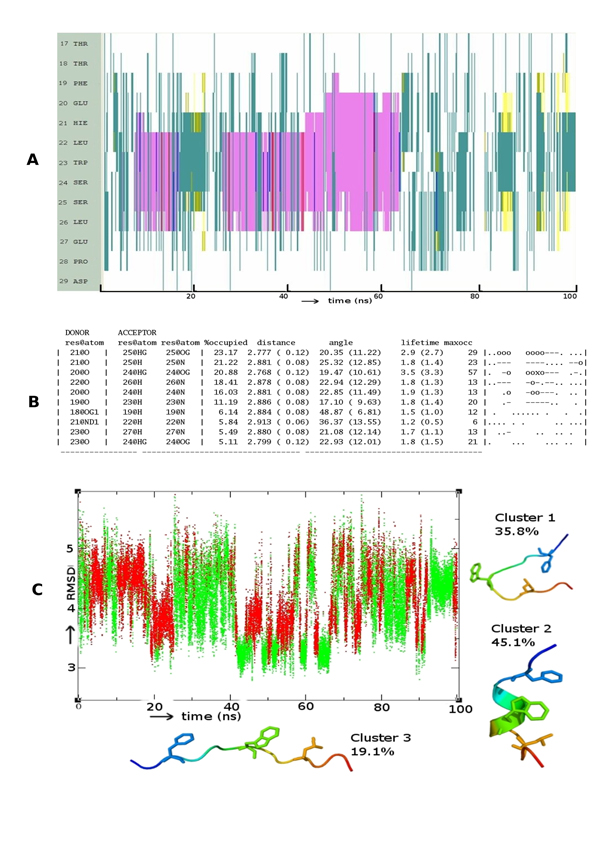
**Folding pattern of p73** Evolution of secondary structures of the p73 peptides as a function of simulation time Colour code: purple, α-helix; red, π-helix; yellow, β-sheet; green, isolated bridge; cyan, turn; white, random coil. (B) Hydrogen bond statistics of the secondary structures averaged over 100 ns of simulations; the lifetime of hydrogen bonds in 5 ns windows is shown as: Space ( ) for 0-5%, dot (.) for 5-20%, dash (-) for 20-40%, o for 40-60%, x for 60-80%, star (*) for 80-95% and at (@) for 95 – 100%. (C) Cluster analysis of secondary structures in terms of RMSD as a function of simulation time; a representative structure (N-terminus in blue, C-terminus in red) from each cluster is shown with % of population;  colour code of the plot: red is helix, yellow is β-Sheet and green is random structure. Conserved residues F19, W23 and L26 are shown as sticks.

 Figure 3A shows that α-helix formation in p73 lies in-between that of p53 and p63. While the helix initially is stable only beyond residue H21 as in p63 (equivalent residue H21), we do find that around 50ns, it extends towards the N-terminal. What is clear is that once this helical state is reached, stability sets in. HB analysis (Figure 3B) shows only one backbone-backbone HB while the other two are side chain-backbone HBs. The occupancy of the backbone-backbone interaction is 18.4% (greater than the 11.8% seen in p63) and 11% between positions F19 and W23. Among the conformations sampled, helices occupy 45% (Figure 3C) and their stability lies in between that of p53 and p63. Clusters 1 and 2 represent disordered motifs.

These observations suggest that there appear to be two factors that are crucial for the formation and persistence of a helix in the TA domain of the p53 family: (a) the kinetics of helix formation which is governed by the nature of the nucleation event that gives rise to a helix and (b) the thermodynamic stability of the helix, once formed. It appears that in all 3 cases, nucleation occurs around residue 22 (L in p53/p73 and I in p63) which is always part of a turn region in these folding studies. The stability of the helix seems to be governed in p53 by the presence of an ionic interaction between D20 and K24. Interestingly this may explain the strong selection seen for the non-contact residue D20 in Mdm2 binding peptides as selected by phage display [47] . The equivalent residues in p63 and p73 are H/D and H/S respectively and it is likely that the lack of such long range interactions may lead to the attenuation of helical stability in these two systems. There is further contribution from the stretch of hydrophobic residues in p53 and p73 i.e., F19, L22, W23, L25 and L26 which tend to cluster hydrophobically. Such patterns of folding nucleated by leucines and persistence of hydrophobic clusters have also been reported in other simulation studies that have investigated this region [48] . Indeed, it seems that the presence of F at position 25 in p63 tends to enhance this aggregation by forming pi-pi interactions with F19 which appears to limit helical formation. Another long range interaction that appears to limit helicity in both p63 and p73 is that mediated between Q20, D24 and Q28 in p63 and between E20, S24 and E27 in p73. Indeed, p53 also has a larger number of backbone HBs and they have longer lifetimes than in p63 and in p73; p73 in turn has more HBs than p63. Together these seem to point towards similarities in helical propensities of p53 and p73 and explain the low helical propensity of the p63 peptides. This may partly explain why only p53 and p73 are involved in similar biological functions [49,50] and the low affinity of MDm2 for p63.

We see that the conformational landscape of the family members has, in addition to the α-helix, other secondary structure elements like β-hairpins (yellow lines in Figure 1A), and π-helix (red lines in Figure 2A). Indeed a beta-hairpin peptide and a cyclic nucleotide with the 3 residues displayed on the same side has been shown to bind to MDM2 [51,52], as have several peptidomimetics [53,54]. In fact it is clear from Figures 1C and 3C that there are several nonhelical conformations of p53 and p73 which have at least two of the 3 residues pointing in the same direction. This is largely missing in p63 and could be another reason why p63 does not seem to interact with MDM2 [55] and MDMX [56].

It is heartening to see that the implicit solvent model maps the conformational landscape of p53 that mirrors the essential features that have been captured in detailed simulations in explicit solvent environments [57]. Both studies demonstrate the existence and persistence of very similar patterns of helicity together with the other structural motifs. Moreover, these are also in accord with experimental studies [3,14,58]. This agreement with experimental and simulation data gives us confidence in the methods that have been applied in our study and the associated findings.

We next attempt to understand the roles of specific residues in modulating the conformational landscapes of the 3 peptides.

### Folding patterns in variants of p53, p63 and p73

Thus far, in summary, there appear to be two major determinants of the folding patterns of the TA domain peptides of the p53 family: nucleation largely by hydrophobic interactions (a kinetic determinant) and stability induced by ion-pairs or long range electrostatics (thermodynamic determinants). We investigate these further by *in-silico* mutagenesis of residues that seem to mediate these interactions and study their folding patterns. Our strategy is to mutate residues in each wild type peptide to their equivalent residues in the other peptides, particularly when we see a difference in their folding patterns; so for example a particular residue in wild type p53 is mutated to the corresponding residue in wild type p63 and *vice versa*.

#### Nucleation site mutants

Detailed examinations of the p53 trajectory (Movie S1 – url found in the Appendix section) suggest that interactions of residue L22 nucleate the formation of the helix (in contrast to the T18-D20 HB) [59,60]. This position is occupied by L in p53 and in p73 and by I in p63. In the 3 simulations we observe that a turn seems to form at this residue and is associated with an HB between the side chain of residue D21 and the backbone of residue W23 (Figure 1D). So we mutated L22 to I in p53 and p73, and I22 to L in p63.

**Figure 4:**
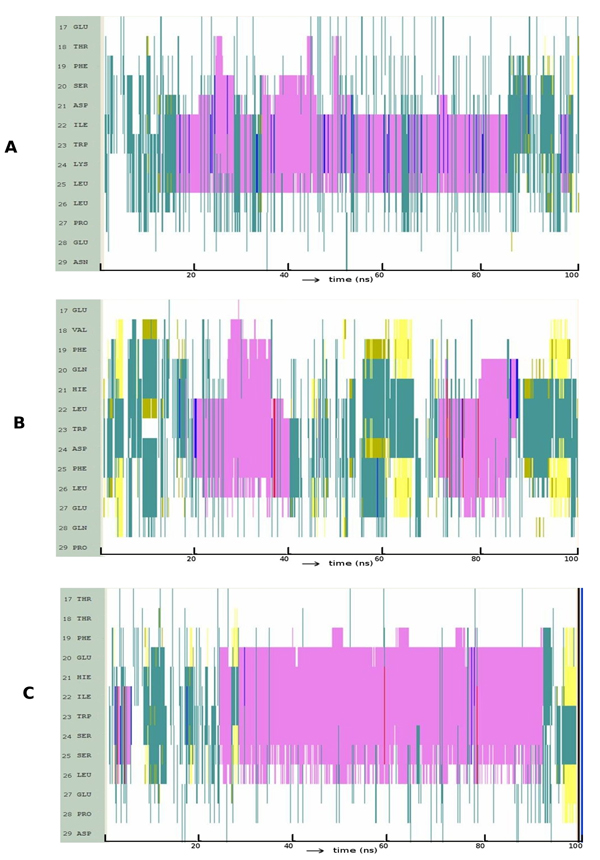
**Evolution of secondary structures of the peptide variants at position 22 along the simulation: **(A) p53: L22I; (B) p63: I22L; (C) p73: L22I; Colour code: purple, α-helix; red, π-helix; yellow, β-sheet; green, isolated bridge; cyan, turn; white, random coil.

In p53, the L to I change disfavors helicity; indeed the helix seems to get truncated at position 22 (Figure 4A). In p63, the I22L change leads to an extension of the helical conformation both spatially and temporally (Figure 4B). The L22I change in p73 seemed to favor a longer and much more stable helix compared to both the p73 wild type and to the L22I mutant of p53 (Figure 4C). L22I in p73 creates a larger space for F19 and L26 (Movie S2) that leads to more packing amongst these hydrophobic residues; this leads to smaller fluctuations and hence results in a more stable helix.

#### Ion-pair mutants

**Figure 5:**
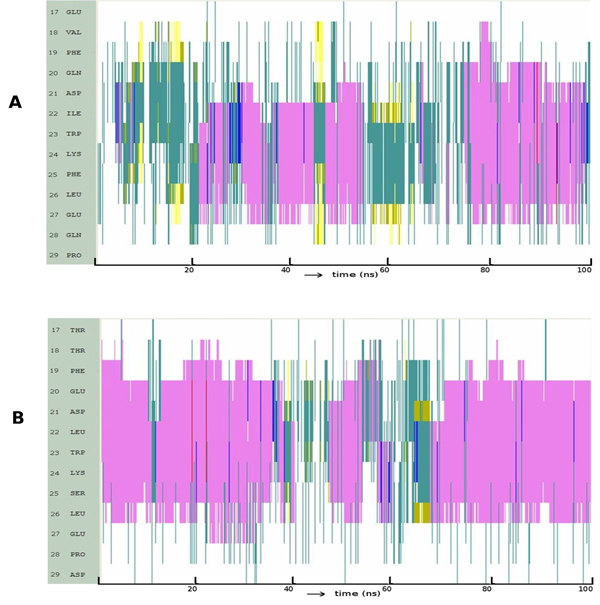
**Evolution of secondary structures of the peptide variants at position 21 and 24 along the simulation:** (A) p63: H21D, D24K; (B) p73: H21D, S24K; Colour code: purple, α-helix; red, π-helix; yellow, β-sheet; green, isolated bridge; cyan, turn; white, random coil.

We earlier described the D21-K24 ion-pair as possibly contributing to the stability of p53. We introduced this ion pair in p63 and p73 at the equivalent positions and found that both the length of the helix and its stability were significantly increased (Figure 5, Movie S3).

The above two observations lead us to then interrogate the significance of this region and ask if this region as a whole may be responsible for the enhanced helicity in wild type p53. For this, we retained the essential W23 and incorporated the flanking residues in the p53 sequence into p63 and p73 as follows: HIWD->DIWK for p63 and HLWS->DLWK for p73. It was observed (Figure 5A, 5B) that this certainly enhances the helicity in both p63 and p73. In both cases, this arises directly as a result of the introduction of K at position 24 whose interactions with D at position 21 and E at position 27 stabilize the extent and stability of the helix (Movie S4).

**Figure 6:**
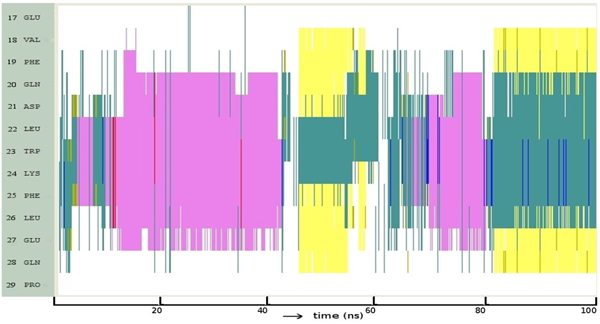
**Evolution of secondary structures of the peptide variants at position 21, 22 and 24 along the simulation; Colour code:** purple, α-helix; red, π-helix; yellow, β-sheet; green, isolated bridge; cyan, turn; white, random coil.

The above observations suggest that the electrostatic modification of p63 and p73 certainly has a significant effect on inducing and stabilizing helicity. To this, we now add the modification whereby we mutated I to L in p63 (taking cues from the differences we identified between p53 and p63 earlier) resulting in p63 with HIWD->DLWK (Movie S5). The long range interaction between D21 and K24 and a cluster of HBs between D21 side chain and back bone amines of W23, K24 and F25 initiate helix formation. Within 2-3ns, the C-terminus becomes fully helical and is stabilized by interactions of K24 with the anionic D21, E27 and the C-terminus (Figure 6). At the same time, at the N-terminus, a hydrophobic cluster comprising of residues F19, L22, F25 and L26 was observed to form and modulate the folding patterns. This hydrophobic cluster, which was quite stable when the L was an I at position 22, hindered the propagation of the helix; upon mutation, this cluster becomes relatively short lived, largely due to the larger fluctuations of L22 compared to those in I22. The ensuing destabilization of the hydrophobic cluster results in a transition of F19 away from F25 and this leads to propagation of the helix at N-terminus. Of course, in p73 we see a similar picture, but now the I22 leads to such propagation (peptide 6, Table 1, Movie S6), thus underlying the complexity of the relationship between sequence and folding patterns.

#### Hydrophobic clusters

As we have seen above, π-π interactions in p63 and in all its variants seem to modulate the folding patterns. In particular, interactions between F19 and F25 in the hydrophobic cluster appear to hinder helical propagation. To examine this, we studied L25F in p53, F25L in p63 and S25F in p73.

**Figure 7:**
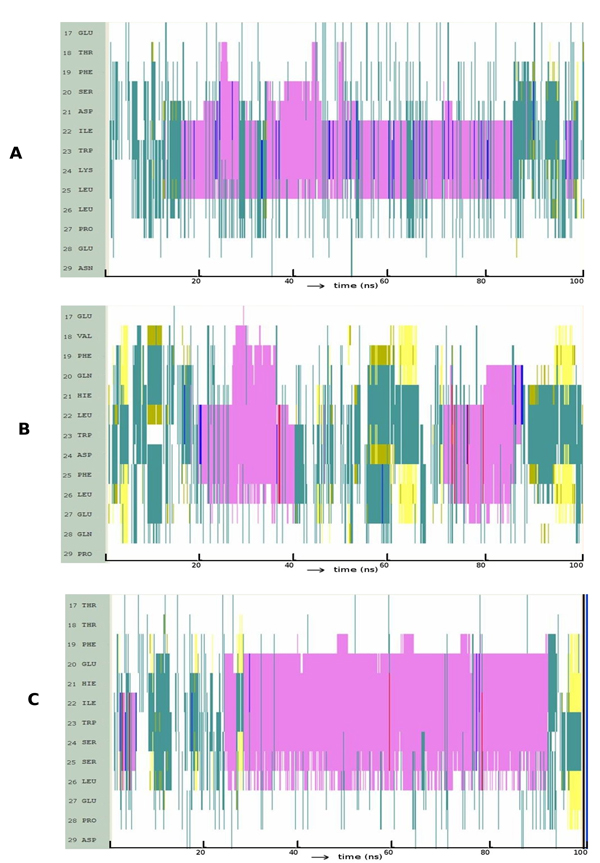
**Evolution of secondary structures of the peptide variants at position 25 along the simulation:** (A) p53: L25F (B) p63: F25L (C) p73: S25F; Colour code: purple, α-helix; red, π-helix; yellow, β-sheet; green, isolated bridge; cyan, turn; white, random coil.

It is clear (Figure 7A, Movie S7) that p53 is severely destabilized, apart from a transient period of helicity as the introduced F collapses against F19 (as seen in wild type p63). In contrast, we see that in p63, the helix is indeed propagated (Figure 7B, Movie S8) due to the F25L mutation including the formation of full length helical structures. The analogous mutation in P73 is S25F and this leads to rapid helix formation followed by its stability (Figure 7C, Movie S9).

### Effect of phosphorylation

Phosphorylation of the TA region of this family controls several of its biologically significant interactions [61] such as interactions with the negative regulator MDM2/MDMX and with the coactivator proteins such as p300 [13]. While the structural implications of this modification on these interactions is understood to some extent for p53 [13,59,60,62,63] little is known about p63 and p73. Having established that our current methodology seems to reproduce experimental data on p53 and rationalize the effects of the differences in sequence amongst the family members, we now examine the effects of phosphorylation on the family members.

**Figure 8:**
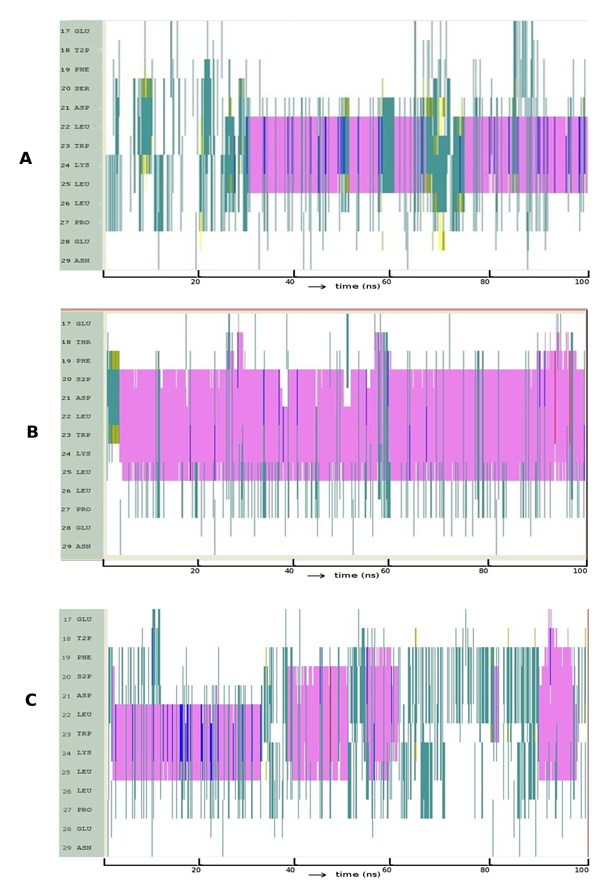
**Evolution of secondary structures of the phosphorylated peptide variants of p53 at (A) T18 (B) S20 and (C) T18 and S20;** Colour code: purple, α-helix; red, π-helix; yellow, β-sheet; green, isolated bridge; cyan, turn; white, random coil.

The region of p53 investigated here has two phosphorylation sites - T18 and S20. Phosphorylation of T18 resulted in some loss of helical propensity (Figure 8A) compared to that in unphosphorylated p53 and this largely seems to arise because of long range interactions between the phosphate and K24 that prevent helical propagation (Movie S10). However helicity is still retained, albeit reduced, and is in accord with our earlier findings [63]. In contrast, phosphorylation of S20 actually enhances the helical propensity of p53 (Figure 8B). This appears to be stabilized by interactions between the phosphate and K24. The reason why this interaction stabilizes the helix appears to be the spatial proximity of these two in contrast to the case of phosphorylated T18. These folding patterns are consistent with the binding affinity of p53 to MDM2 where only the phosphorylation of T18 attenuates binding to MDM2 [18]. Indeed, the phosphorylation of S20 needs a helical conformation as this appears to be a structural requirement for binding to p300 [64]. When both T18 and S20 are phosphorylated the folding pattern (Figure 8C) seemed to display an initial effect of phosphorylation of T18 (as in Figure 8A) followed by that of S20 (Figure 8B). Consistent with the individual phosphorylations, the pattern of interactions seen upon double phosphorylation is conserved. Recent work has demonstrated that p53 has to be significantly helical to optimally interact with p300 [62] and perhaps even for simultaneous binding to MDM2 and p300 [13]. Our observations also suggest that double phosphorylation does retain the helicity required for binding to p300 and that dephosphorylation of T18 is not required prior to phosphorylation of S20 for such binding events.

**Figure 9:**
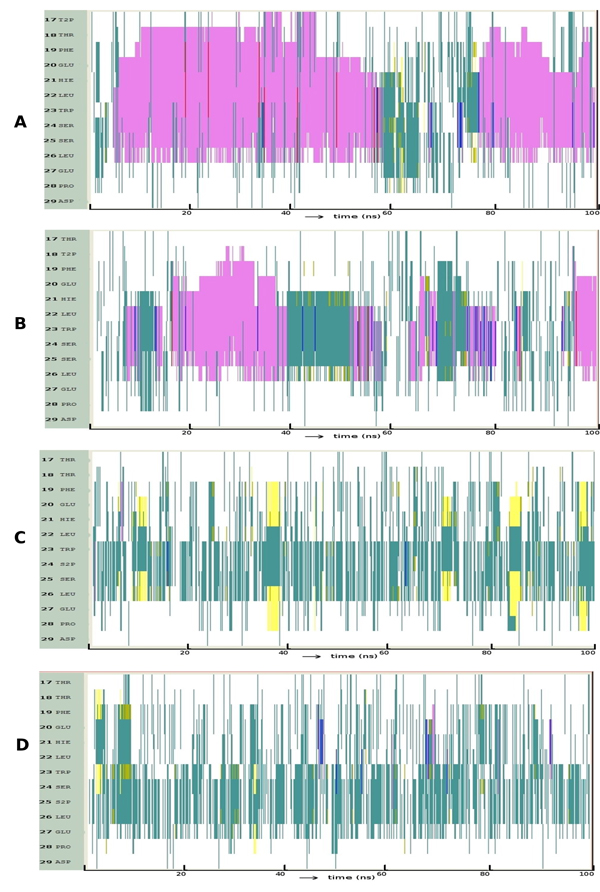
**Evolution of secondary structures of the phosphorylated peptide variants of p73 at (A) T17 (B) T18 (C) S24 and (D) S25;** Colour code: purple, α-helix; red, π-helix; yellow, β-sheet; green, isolated bridge; cyan, turn; white, random coil.

In the case of p73, there are four potential phosphorylation sites – T17, T18, S24 and S25 (Table 1, Sequence 3). Phosphorylation at T17 and T18 increases the helicity much more compared to that seen in unphosphorylated p73 (Figure 9A – 9B). This latter is known to be required for binding of p73 to p300 [62] and our model suggests that the picture is somewhat similar to that seen in p53. Further, our models correlate well with the experimental finding that phosphorylated p73 binds to MDM2 two fold better than does phosphorylated p53 (Compare Figure 8A with Figure 9B). Interestingly phosphorylations at S24 and S25 show no helicity at all (Figure 9C – 9D) and may suggest modifications either for dissociations of p73 from partners or for associations with other proteins; experimental data on these is not available yet. Long range repulsive interactions between the developing anionic environment as a result of phosphorylation and the acidic residues E20, E27 seems to be responsible for the lack/loss of helicity. It appears that position T18 in p53 and positions S24 and S25 in p73 are switches that control the helical nature of the peptide.

## Effects of explicit solvent

**Figure 10:**
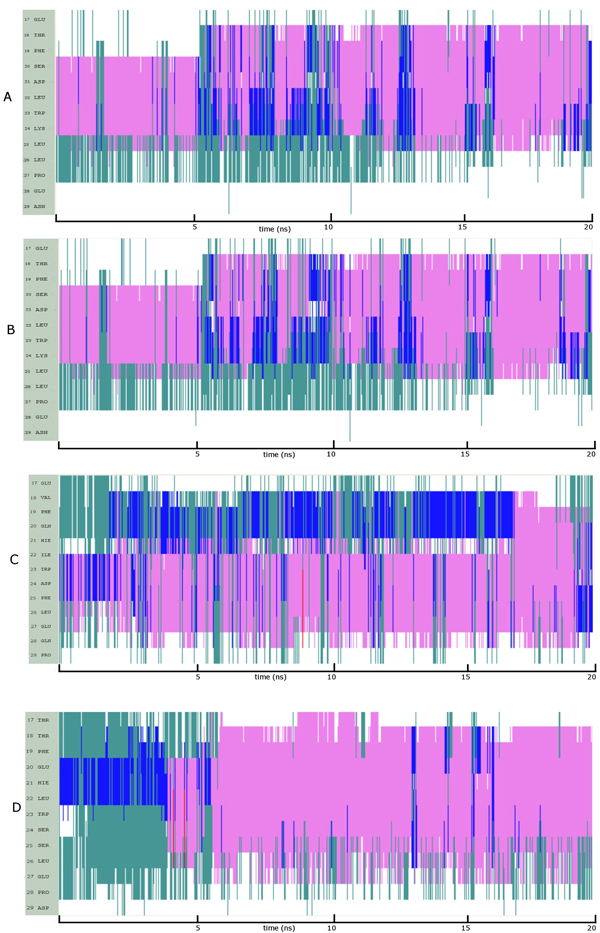
**Evolution of secondary structures in explicit water of (A) p53:** nucleation point (B) p53: ionic interaction (C) p63: nucleation point and (D) p73: nucleation point; Colour code: purple, α-helix; red, π-helix; yellow, β-sheet; green, isolated bridge; cyan, turn; white, random coil.

In order to test the robustness of these simulations, we extracted snapshots along the trajectories describing the folding of p53, p63 and p73 and carried out simulations on these in explicit water. The snapshots were chosen to represent the states that we have identified as nucleation points. For p53, we additionally examine a state which was characterized by an ionic interaction between D21 and K24 to examine the stability of this salt bridge. We see that p53 readily folds into a helix in both cases (Figure 10A, Figure 10B), as does p73 (Figure 10D), albeit somewhat less readily. In contrast, p63 folds (Figure 10C) into a helix very late in our simulation, but nevertheless folds. The salt bridge between D21 and K24 in p53 appears to “catalyzes” the rapid onset of helicity (Movie S11 and Movie S12) and is non-existent in p63 and p73. The delay in p63 is due to the stable hydrophobic cluster (Movie S13).

Together these data show that the implicit solvent model captures the essential features of the folding patterns of these sequences although it is clear from the p63 data that explicit solvent does mediate barrier crossings that enable the transition from the cluster to the folded helix [65].

Finally, although all 3 fold into helices, and indeed p73 seems to form a longer helix (Movie S14), there are two characteristics that can be used to understand why the order of binding is p53>p73>p63. The first is that MDM2 modulates the binding of these peptides as has been shown elsewhere [20,38]. The second feature is the observation in the current study that while p53 forms a helix rapidly (see for example Figure 10A), the onset is delayed in p73 (Figure 10D) and only occurs very late in the simulation in p63 (Figure 10C).

## Conclusions

Folding studies of the TA domain of the p53 family in an implicit solvent model support earlier findings that p53 TF is intrinsically largely unstructured with some regions of helicity and an overall high propensity for helical structure. This helix is responsible for mediating the interactions of these proteins with a range of partners. Helical propensity is similar between p53 and p73, but is very low for p63. Mutations suggest that electrostatic interactions mediated by K24 are important for the thermodynamic stability of the helix, whose nucleation and kinetics are in turn controlled by the size of the hydrophobic residue at position 22 (stabilizing hydrophobic clusters). Successful benchmarks against available experimental/computational data for p53 enabled us to suggest mutations in peptides derived from p63 and p73 that could bind effectively to MDM2. Further, we find that the effects of phosphorylations (perturbation of helicity due to phosphorylation of T18 and enhancement of helicity due to phosphorylation of S20) are in accord with experimental findings and suggest that the helical propensity of phosphorylated S20 in p53 is very high. This enables doubly phosphorylated p53 (at T18 and S20) to bind to p300 without the need for dephosphorylating T18. Our results also are in accord with the thesis that p53 may exist as a ternary complex in unstressed cells with MDM2 and with p300 and that stress related phosphorylation of T18 and S20 will enable dissociation of MDM2 and tighter association with p300 – an event directly linked to enhanced helicity. This also corroborates our earlier finding [59,60,63] that phosphorylation of T18 does not diminish helicity of TA to the extent that it should disrupt binding to MDM2; indeed the disruption of binding was attributed to the development of electrostatic repulsions between phosphorylated T18 and the MDM2 surface and our current results are in accord with our earlier hypothesis [17]. Phosphorylation of p73 at T17 and T18 enhances the helicity much more than at equivalent position in p53 and may be responsible for the higher affinity of phosphorylated p73 for MDM2 and for p300 [62]. Similarities in helicity in p53 and p73 may be one of the reasons why p73 was able to replace p53 in p53-deficient breast cancer cells [66]. This also may have implications for the differential regulation of post-translationally modified p53 and p73. For example p53 is stabilized by releasing it from sequestration by MDM2 while p73-dependant transcription is abrogated (perhaps by tighter binding to MDM2) during events such as mitosis [67].

Finally our results also point towards some resolution of the controversy over the interaction between MDM2 and p63 [36,37]. It is clear that under conditions such as an excess of MDM2 and/or low concentrations of p53, the interaction of MDM2 with p63 occurs [36]. This is in line with in-vitro observations that do suggest weak interactions between the two [68] . Together, the fact that the FXXXWXXL motif is shared by p53, p63 and p73 and this pattern is recognized by MDM2 suggests that MDM2 should bind to all 3 proteins. Our results show that the hydrophobic cluster that forms in p63 “slows” down folding but yet eventually does lead to a conformation that is suitable for sequestration by MDM2; this may partly be responsible for the experimental demonstrations of weak interaction between p63 and MDM2 (relative to the interactions between MDM2 and p53/p73). This possibly may account for the transient nature of this complex and hence evades trapping [36]. In addition, as we have shown elsewhere, the dynamic surface of MDM2 modulates the interactions with these peptides [20,38]. While there is always the possibility of additional protein players in these orchestrated interactions, nevertheless, the current observations certainly show that the intrinsic propensity of these sequences determine their abilities to fold into states that can interact with modulator proteins such as MDM2, with varying affinities and this in turn will impinge upon their biological functions.

## Competing interests

The authors declare that they have no competing interests.

## Authors' contributions

JM: Simulations, analysis of simulation data, mutation ideas, drafting, drafting figures and movie production and editing of the manuscript.

MA: Simulations, analysis of simulation data, mutation ideas, drafting and editing of the manuscript.

RB: Discussion, suggestions.

DL: Discussion, suggestions.

CV: Analysis of simulation data, mutation ideas, editing of manuscript, discussion and suggestions.

## Appendix

Movies, Movie S1 – S14, in mpeg format are available for download from internet web page http://web.bii.a-star.edu.sg/~jagadeesh/p53/

## References

[B1] Harris CC p53 Tumor suppressor gene: from the basic research laboratory to the clinic-an abridged historical perspective. Carcinogenesis 1996 17 1187 1198 10.1093/carcin/17.6.1187 8681432

[B2] El-Deiry W Regulation of p53 downstream genes. Semin Cancer Biol 1998 8 345 357 10.1006/scbi.1998.0097 10101800

[B3] Bell S Klein C Muller L Hansen S Buchner J p53 contains large unstructured regions in its native state. J Mol Biol 2002 322 917 927 10.1016/S0022-2836(02)00848-3 12367518

[B4] Levine AS Kelly K Recruitment of p300:CBP in p53-dependent signal pathways. Cell 1997 89 1175 1184 10.1016/S0092-8674(00)80304-9 9215639

[B5] Lill NL Grossman SR Ginsberg D DeCaprio J Livingston DM Binding and modulation of p53 by p300: CBP coactivators. Nature 1997 387 823 827 10.1038/42981 9194565

[B6] Thut CJ Chen JL Klemm R Tjian R p53 transcriptional activation mediated by coactivators TAFII40 and TAFII60. Science 1994 267 100 104 10.1126/science.7809597 7809597

[B7] Haupt Y Maya R Kazaz A Oren M Mdm2 promotes the rapid degradation of p53. Nature 1997 387 296 299 10.1038/387296a0 9153395

[B8] Kubbutat MH Jones SN Vousden KH Regulation of p53 stability by Mdm2. Nature 1997 387 299 303 10.1038/387299a0 9153396

[B9] Joerger AC Fersht AR Structural biology of the tumor suppressor p53. Annu Rev Biochem 2008 77 557 82 10.1146/annurev.biochem.77.060806.091238 18410249

[B10] Danovi D Meulmeeste E Pasin D Migliorini D Capra M Frenk R de Graaf P Francoz S Gasparini P Gobbi A Helin K Pelicci PG Jochemsen AG Marine JC Amplification of mdmx (or mdm4) directly contributes to tumor formation by inhibiting p53 tumor suppressor activity. Mol Cell Biol 2004 24 13 5835 5843 10.1128/MCB.24.13.5835-5843.2004 15199139 PMC480894

[B11] Toledo F Wahl GM Mdm2 and mdm4: p53 regulators as targets in anticancer therapy. Int J Biochem Cell Biol 2007 39 7-8 1476 1482 10.1016/j.biocel.2007.03.022 17499002 PMC2043116

[B12] Pazgier M Liu M Zou G Yuan W Li C Li C Li J Monbo J Zella D Tarasov SG Lu W Structural basis for high-affinity peptide inhibition of p53 interactions with MDM2 and MDMX. Proc Natl Acad Sci U S A 2009 106 12 4665 70 10.1073/pnas.0900947106 19255450 PMC2660734

[B13] Ferreon JC Lee CW Arai M Martinez-Yamout MA Dyson HJ Wright PE Cooperative regulation of p53 by modulation of ternary complex formation with CBP/p300 and HDM2. PNAS 2009 106 6591 6596 10.1073/pnas.0811023106 19357310 PMC2672497

[B14] Lee H Mok KH Muhandiram R Park KH Suk JE Kim DH Chang J Sung YC Choi KY Han KH Local structural elements in the mostly unstructured transcriptional activation domain of human p53. J Biol Chem 2000 275 38 29426 32 10.1074/jbc.M003107200 10884388

[B15] Li C Liu M Monbo J Zou G Li C Yuan W Zella D Lu WY Lu W Turning a scorpion toxin into an antitumor miniprotein. J Am Chem Soc 2008 130 41 13546 13548 10.1021/ja8042036 18798622 PMC3810402

[B16] Haizhen Zhong Heather A Carlson. Computational studies and peptidomimetic design for the human p53-MDM2 complex. Prot Struct Func Bioinformatics 2005 58 222 234 10.1002/prot.20275 15505803

[B17] Brown CJ Srinivasan D Jun HJ Coomber D Verma CS Lane DP The electrostatic surface of MDM2 modulates the specificity of its interaction with phosphorylated and unphosphorylated p53 peptides. Cell Cycle 2008 7 608 610 18256546 10.4161/cc.7.5.5488

[B18] Schon O Friedler A Bycroft M Freund SMV Fersht AR Molecular mechanism of the interaction between MDM2 and p53. J 2002 323 491 501 10.1016/S0022-2836(02)00852-5 12381304

[B19] Kutchukian PS Yang JS Verdine GL Shakhnovich EI All-atom model for stabilization of alpha-helical structure in peptides by hydrocarbon staples. J Am Chem Soc, 2009 131 13 4622 4627 10.1021/ja805037p PMC2735086 19334772

[B20] Dastidar SG Lane DP Verma CS Multiple peptide conformations give rise to similar binding affinities: molecular simulations of p53-MDM2. J Am Chem Soc 2008 130 41 13514 5 10.1021/ja804289g 18800837

[B21] Zondlo SC Lee AE Zondlo NJ Determinants of Specificity of MDM2 for the Activation Domains of p53 and p65: Proline27 Disrupts the MDM2-binding motif of p53. Biochemistry, 2006 45 11945 11957 10.1021/bi060309g 17002294

[B22] Bernal F Tyler AF Korsmeyer SJ Walensky LD Verdine GL Reactivation of the p53 tumor suppressor pathway by a stapled p53 peptide. J Am Chem Soc 2007 129 9 2456 2457 10.1021/ja0693587 17284038 PMC6333086

[B23] Yang A Schweitzer R Sun D Kaghad M Walker N Bronson RT Tabin C Sharpe A Caput D Crum C McKeon F p63 is essential for regenerative proliferation in limb, craniofacial and epithelial development. Nature, 1999 398 714 718 10.1038/19539 10227294

[B24] Mills AA Zheng B Wang XJ Vogel H Roop DR Bradley A p63 is a p53 homologue required for limb and epidermal morphogenesis. Nature 1999 398 708 713 10.1038/19531 10227293

[B25] Barbieri CE Pietenpol JA p63 and epithelial biology. Exp. Cell Res 2006 312 695 706 10.1016/j.yexcr.2005.11.028 16406339

[B26] Koster M.I Roop D.R p63 and epithelial appendage development. Differentiation 2004 72 364 370 10.1111/j.1432-0436.2004.07208002.x 15606495

[B27] Van Bokhoven H McKeon F Mutations in the p53 homolog p63: allele-specific developmental syndromes in humans. Trends Mol. Med 2002 8 133 139 10.1016/S1471-4914(01)02260-2 11879774

[B28] Levrero M De Laurenzi V Costanzo A Sabatini S Gong J Wang JYJ Melino G The p53/p63/p73 family of transcription factors: overlapping and distinct functions. J Cell Sci 2000 113 1661 1670 10769197 10.1242/jcs.113.10.1661

[B29] Kaghad M Bonnet H Yang A Creancier L Biscan JC Valent A Minty A Chalon P Lelias JM Dumont X Ferrara P McKeon F Caput D Monoallelically expressed gene related to p53 at 1p36, a region frequently deleted in neuroblastoma and other human cancers. Cell 1997 90 809 819 10.1016/S0092-8674(00)80540-1 9288759

[B30] Yang A Kaghad M Wang Y Gilleett E Fleming MD Dotsch V Andrews NC Caput D McKeon F p63, a p53 homologue at 3q27-29, encodes multiple products with transactivating, death-inducing, and dominant-negative activities. Mol. Cell 1998 2 305 316 10.1016/S1097-2765(00)80275-0 9774969

[B31] p53 Knowledge base http:// http://p53.bii.a-star.edu.sg

[B32] IARC TP53 mutation database http://www-p53.iarc.fr/

[B33] Gressner O Schilling T Lorenz K Schulze Schleithoff E Koch A Schulze Bergkamen H Maria Lena A Candi E Terrinoni A Catani MV Oren M Melino G Krammer PH Stremmel W Muller M Tap63[alpha] induces apoptosis by activating signaling via death receptors and mitochondria. EMBO J 2005 24 2458 2471 10.1038/sj.emboj.7600708 15944736 PMC1173149

[B34] Melino G Lu X Gasco M Crook T Knight RA Complexities in the functional regulation of p63 and p73: from development to cancer? Trends in Biological Sciences 2003 28 663 670 10.1016/j.tibs.2003.10.004 14659698

[B35] Haupt Y p53 Regulation: a family affair. Cell Cycle 2004 3 7 884 5 15190198 10.4161/cc.3.7.996

[B36] Calabrò V Mansueto G Parisi T Vivo M Calogero RA La Mantia G The human MDM2 oncoprotein increases the transcriptional activity and the protein level of the p53 homolog p63. J Biol Chem 2002 277 4 2674 2681 10.1074/jbc.M107173200 11714701

[B37] Ying H Chang DL Zheng H McKeon F Xiao ZX DNA-binding and transactivation activities are essential for TAp63 protein degradation. Mol Cell Biol 2005 25 14 6154 6164 10.1128/MCB.25.14.6154-6164.2005 15988026 PMC1168832

[B38] Madhumalar A Lee HJ Brown CJ Lane D Verma C Design of a novel MDM2 binding peptide based on the p53 family. Cell Cycle 2009 8 17 2828 36 19713735 10.4161/cc.8.17.9516

[B39] Fry D Vassilev L Targeting protein–protein interactions for cancer therapy. Journal of Molecular Medicine, 2005 83 12 955 963 10.1007/s00109-005-0705-x 16283145

[B40] Case DA Cheatham TE Darden T Gohlke H Luo R Merz KM Onufriev A Simmerling C Wang B Woods RJ The amber biomolecular simulation programs. J Comput Chem 2005 26 1668 1688 10.1002/jcc.20290 16200636 PMC1989667

[B41] Shell SM Ritterson R Dill KA A test on peptide stability of amber force fields with implicit solvation. J Phys Chem B, 2008 112 22 6878 6886 10.1021/jp800282x PMC2699260 18471007

[B42] Onufriev A Bashford D Case DA Exporling protein native states and large-scale conformational changes with a modified generalized Born model. Proteins 2004 55 383 394 10.1002/prot.20033 15048829

[B43] van Gunsteren WF Berendsen HJC Algorithms for macromolecular dynamics and constraint dynamics. Molecular Physics, 1977 34 5 1311 1327 10.1080/00268977700102571

[B44] Jorgensen W Chandrasekhar J Madura J Comparison of simple potential functions for simulating liquid water. J. Chem. Phys 1983 79 926 935 10.1063/1.445869

[B45] Humphrey W Dalke A Schulten K VMD: visual molecular dynamics. Journal of Molecular Graphics 1996 14 33 38 10.1016/0263-7855(96)00018-5 8744570

[B46] Feig M Karanicolas J Brooks CL Mmtsb tool set: enhanced sampling and multiscale modeling methods for applications in structural biology. J Mol Graph Model, 2004 22 5 377 395 10.1016/j.jmgm.2003.12.005 15099834

[B47] Böttger V Böttger A Howard SF Picksley SM Chène P Garcia-Echeverria C Hochkeppel HK Lane DP Identification of novel mdm2 binding peptides by phage display. Oncogene 1996 13 10 2141 2147 8950981

[B48] Espinoza-Fonseca LM Leucine-rich hydrophobic clusters promote folding of the N-terminus of the intrinsically disordered transactivation domain of p53. FEBS Lett, 2008 583 556 560 10.1016/j.febslet.2008.12.060 19162020

[B49] Billon N Terrinoni A Jolicoeur C McCarthy A Richardson WD Melino G Raff M Roles of p53 and p73 during oligodendrocyte development. Development, 2004 131 6 1211 1220 10.1242/dev.01035 14960496

[B50] Cui R Nguyen TT Taube JH Stratton SA Feuerman MH Barton MC Family members p53 and p73 act together in chromatin modification and direct repression of α-fetoprotein. J Biol Chem, 2005 280 39152 39160 10.1074/jbc.M504655200 16203738

[B51] Fasan R Dias RL Moehle K Zerbe O Obrecht D Mittl PR Grütter MG Robinson JA Structure-activity studies in a family of beta-hairpin protein epitope mimetic inhibitors of the p53-HDM2 protein-protein interaction. Chembiochem 2006 7 3 515 26 10.1002/cbic.200500452 16511824

[B52] Secchiero P Melloni E Tiribelli M Gonelli A Zauli G Combined treatment of CpG-oligodeoxynucleotide with nutlin-3 induces strong immune stimulation coupled to cytotoxicity in B-chronic lymphocytic leukemic (B-CLL) cells. J Leukoc Biol, 2008 83 434 437 10.1189/jlb.0707459 17998303

[B53] Robinson JA Beta-hairpin peptidomimetics: design, structures and biological activities. Acc Chem Res 2008 41 10 1278 88 10.1021/ar700259k 18412373

[B54] Grässlin A Amoreira C Baldridge KK Robinson JA Thermodynamic and Computational Studies on the Binding of p53-Derived Peptides and Peptidomimetic Inhibitors to HDM2. Chembiochem 2009 10 8 1360 1368 10.1002/cbic.200900008 19408261

[B55] Ying H Chang DL Zheng H McKeon F Xiao ZX DNA-binding and transactivation activities are essential for TAp63 protein degradation. Mol Cell Biol 2005 25 14 6154 64 10.1128/MCB.25.14.6154-6164.2005 15988026 PMC1168832

[B56] Wang XQ Arooz T Siu WY Chiu CHS Lau A Yamashita K Poon RYC MDM2 and MDMX can interact differently with ARF and members of the p53 family. FEBS Letters, 2001 490 202 208 10.1016/S0014-5793(01)02124-X 11223036

[B57] Espinoza-Fonseca LM Trujillo-Ferrara JG Transient stability of the helical pattern of region F19-L22 of the N-terminal domain of p53: A molecular dynamics simulation study. Biochem Biophys Res Commun 2006 343 110 116 10.1016/j.bbrc.2006.02.129 16530164

[B58] Botuyan MV Momand J Chem Y Solution conformation of an essential region of the p53 transactivation domain. Fold Des 1997 2 331 342 10.1016/S1359-0278(97)00047-3 9427007

[B59] Brown CJ Srinivasan D Jun HJ Coomber D Verma CS Lane DP The electrostatic surface of MDM2 modulates the specificity of its interaction with phosphorylated and unphosphorylated p53 peptides. Cell Cycle 2008 7 608 610 18256546 10.4161/cc.7.5.5488

[B60] Dodson GG Lane DP Verma CS Molecular simulations of protein dynamics: new windows on mechanisms in biology. EMBO reports 2008 9 144 150 10.1038/sj.embor.7401160 18246106 PMC2246404

[B61] Kruse JP Gu W Modes of p53 regulation. Cell, 2009 137 609 622 10.1016/j.cell.2009.04.050 PMC3737742 19450511

[B62] Burge S Teufel DP Townsley FM Freund SMV Bycroft M Fersht AR Molecular basis of the interactions between the p73 N terminus and p300: Effects on transactivation and modulation by phosphorylation. PNAS 2009 106 3142 3147 10.1073/pnas.0900383106 19218448 PMC2651247

[B63] Lee HJ Srinivasan D Coomber D Lane DP Verma CS Modulation of the p53-MDM2 interaction by phosphorylation of Thr18: a computational study. Cell Cycle 2007 6 2604 2611 17957142 10.4161/cc.6.21.4923

[B64] Dornan D Shimizu H Perkins ND Hupp TR DNA-dependent acetylation of p53 by the transcription coactivator p300. J Biol Chem, 2003 278 15 13431 41 10.1074/jbc.M211460200 12499368

[B65] Xu F Cross TA Water Foldase activity in catalyzing polypeptide conformational rearrangements. Proc Natl Acad Sci U S A 1999 96 16 9057 9061 10.1073/pnas.96.16.9057 10430894 PMC17731

[B66] Vayssade M Haddada H Faridoni-Laurens L Tourpin S Valent A Benard J Ahomadegbe J p73 functionally replaces p53 in adriamycin-treated, p53-deficient breast cancer cells. Int. J. Cancer 2005 116 860 869 10.1002/ijc.21033 15849742

[B67] Fulco M Costanzo A Merlo P Mangiacasale R Strano S Blandino G Balsano C Lavia P Levrero M p73 is regulated by phosphorylation at the G2/M transition. J Biol Chem 2003 278 49196 49202 10.1074/jbc.M304921200 12920125

[B68] Little NA Jochemsen AG Hdmx and Mdm2 can repress transcription activation by p53 but not by p63. Oncogene 2001 20 33 4576 80 10.1038/sj.onc.1204615 11494153

